# Hospital and Institutional News

**Published:** 1912-08-31

**Authors:** 


### HOSPITAL AND INSTITUTIONAL NEWS.

### THE COMPUNCTION OF THE KINGSTON GUARDIANS.

As a result of ours and other articles on the action of the Kingston-on-Thames Guardians in advertising for a workhouse master and matron, to be held by a married couple, " without encumbrance," some consciousness of the disgrace attaching to the authors of such advertisements has manifested itself, though complete reparation of the offence is yet to seek. On Tuesday the Guardians held a meeting, at which Mrs. Harker, the wife of Dr. Harker, of the National Physical Laboratory at Teddington, brought forward a motion regretting the insertion of the words " without encumbrance " in the advertisement, and binding the Guardians in the future to impose no such condition to the appointment of their officers. The discussion of this motion revealed that the public indignation which has been aroused has had some effect on the Guardians, who on the whole were in favour of Mrs. Harker's resolution; but the realisation of the inhumanity of their action is still incomplete, we fear, since it was felt that on the present occasion the appointments must be made in accordance with the terms of the advertisement. In other words, the Kingston Guardians are content to prolong the stigma which their advertisement has attached to them, for they will remain pilloried as depriving their officers of the rights of paternity and maternity so long as their advertisement is not wholly quashed and repudiated. Married Poor-Law officials should combine to insist on proper provision being provided, and we hope that the successful candidates for the posts now advertised will quickly put the Guardians' professions of regret to the practical test by begetting a family and demanding proper quarters for their upbringing. The public will certainly support them in such action.

### PAYING PATIENTS AND WORCESTER INFIRMARY

The house committee of Worcester General Infirmary is considering a letter from Captain Sherwell, who suggests that every patient should pay a fee of sixpence or a shilling a day instead of receiving treatment free as at present. The question of introducing such payments is never a very easy one, and particularly at the outset, as Mr. Leonard Rea, the secretary-superintendent of King Edward's Hospital, Cardiff, explained in The Hospital some months ago. There are several pitfalls to be avoided. He drew especial attention to the importance of explaining to the patients that the new charges, " namely, 6d. towards the 3s. 6d. per day which represents the average cost of each in-patient's maintenance," were only a small proportion of their actual cost to the institution, and the word " maintenance " was substituted for the word " food " to " make it quite clear that the charge is in no respect made in regard to medical service, which is, of course, given without money and without price." The fact that no charge was made to patients who cannot afford to pay and the machinery for collecting the in-patients' payments were then explained, and the conclusions had peculiar authority as having been arrived at after nine months of experiment. It will be interesting to see what is the decision of the house committee of Worcester General Infirmary; as they are likely to find similar experiments valuable, we refer them to Mr. Leonard Rea's article " Paying Patients at Cardiff Infirmary. How the New System was Instituted," which appeared in The Hospital of December 30, 1911.

### NEW RESEARCH SCHOOL FOR A LONDON CHEST HOSPITAL.

Owing to the munificence of a private donor a school for research and clinical instruction in diseases of the chest has been established in connection with the Royal Hospital for Diseases of the Chest, City Road, London. The new building of the hospital is equipped with pathological and bacteriological laboratories, radiographic and electro-cardiac departments, a pathological museum and lecture hall. The medical school will be opened about the middle of October, and it is intended to issue a full syllabus towards the end of September. Arrangements are being made for demonstrations in recent methods of diagnosis and treatment, including the administration of tuberculin. It is intended that the medical school should become a training centre for those who wish to qualify themselves for sanatorium and tuberculosis dispensary posts. Dr. Barty King is the acting-dean of the school.

### AN IRISH CRITICISM OF MENTAL LEGISLATION.

The recent proposals for the legislative control of the feeble-minded lend additional interest to the remarks on the value of legislation which appear in the report of Dr. M. J. Nolan, resident medical superintendent of Down District Mental Hospital, Downpatrick. He impresses at some length on his committee that " legislation *per se* will not stamp out insanity," and adds: " Practical eugenics should, then, be chiefly concerned with the various factors that precipitate insanity, which are all elements in ordinary social life. The common-sense of a community exercised on such matters can effect more than the experimental labours of laboratory workers." A light, perhaps, is thrown on this from the memorandum of inspection by Dr. W. R. Dawson, which is appended to the report. Dr. Dawson says: " It would appear that a large proportion of the cases suffer from acute first attacks of a curable nature." Nevertheless, the demands for increased facilities for mental research have been growing of late more insistent. If attention is now much directed towards legislation for the mentally afflicted, Dr. Nolan does not omit to remind us of the other aspect of legislation in its relation to the officers of mental hospitals. As president of the Irish Division of the Asylum Workers' Association of Great Britain and Ireland, Dr. Nolan is well entitled to speak, and he congratulates his committee on their curtailment of the hours of duty to seventy per week. He admits further that "the unrest which is so detrimental to efficient service" in mental hospitals is largely due to the indifference of committees to the exceptional strain of mental work and to the great need for those engaged in it to have a due amount of recreation and a pension as the reward for faithful service. In this matter he claims that the Down District Committee of Management, of which Major Blackwood-Price is chairman, have set a good example.

### EDINBURGH'S NEW PROFESSOR.

Professor J. Lorrain Smith, of Manchester, has been appointed Professor of Pathology to the University of Edinburgh in succession to Professor Greenfield. The record of Professor Lorrain Smith is an interesting one. He graduated at Edinburgh in both medicine and arts; and engaged in post-graduate studies at Oxford and Cambridge, and at the latter held the John Lucas Walker Studentship in Pathology. At Cambridge he afterwards became demonstrator of pathology; and then lecturer and professor of pathology at Queen's College, Belfast, where he was also honorary pathologist to the Royal Victoria Hospital. On moving to Manchester as professor in 1904 he held a similar appointment to the Manchester Royal Infirmary. He is a Fellow of the Royal Society and has published numerous papers on pathology.

### POST-GRADUATE WORK AT THE CONGRESS.

A special meeting for the discussion of post-graduate study in medicine will be held during the sessions of the International Congress of Medicine in London next year under the auspices of the International Committee for Post-Graduate Medical Education. The programme of the meeting includes papers dealing with special and general aspects of post-graduate study. A feature will be the reports on the collective researches instituted by the committee into the conditions and nature of medical study in all civilised countries. Three special subjects or themes have been selected for debate and report. These are (1) present methods of University education in medicine, with reference to the diversities among and the legal status of various degrees and curricula; (2) social medicine as a special subject for the curriculum; and (3) general suggestions for reform and improvement of the courses, both for post-graduates and for ordinary medical students. The International Committee has worked hard and patiently since its constitution, and we understand that the reports are full and clear, so that they will be of far greater general interest than the somewhat one-sided reviews, such as the Flexner memorandum. By the way, the committee has not yet found a successor to the late Dr. Pavy, one of the English representatives. The English members of the committee are appointed, we believe, by the Secretary of State for Foreign Affairs, and none of them (as we pointed out at the time when the first appointments were made) can be said to be authorities on the subject of graduate study. It is to be hoped that next year's Congress will see some effort made to put these appointments in the hands of either the General Medical Council or the responsible colleges, with due regard to the post-graduate institutions that are already doing such good work in this country.

### CAUSES OF FRICTION AND HOSPITAL VISITORS.

The hospital secretary or committeeman is a man who quickly learns not to despise detail, for details are more frequent causes of friction than anything else. From time to time we have to chronicle in these columns causes of friction which sometimes produce unfortunate results apparently out of all proportion to their origin. The question "Who should have charge of the dispensary keys?" was a recent case in point. A more general cause is great disparity of age or standing between two such chief officials as a secretary and a matron. When the latter's term of office has been long and uninterrupted, the resignation or death of her colleague, and the new appointment to the secretaryship which it necessitates is sometimes taken advantage of by an elderly matron whose discretion has been blurred by a long term of office. Where this is the case the path of the new secretary becomes thorny in the extreme, and peace and smooth working can generally be secured only by the elder official's resignation. Another cause of friction is sometimes found in the position of the hospital visitor, a post for which great tact is required if useful work is to be done without interference with ward routine. It is sometimes suggested that visitors should be provided with "passes," as official intimation of their position, though many will think such a formality unnecessary. What is our readers' experience on this point?

### A MEDICAL CENTENARIAN.

Since the medical profession does not stand very high in the category of those the members of which are long lived, every case of a medical centenarian has an interest. The latest example is that of Dr. Edgar Jones, of Great Burstead, near Billericay, who has just died at the great age of 102. He qualified in 1834, practised near Briston and at Saffron Walden, but retired in 1846, when he went to live at Great Burstead. Six years later he was placed on the Commission of the Peace, and only last March the Essex Court of Quarter Sessions passed a resolution congratulating him on having sat on the bench for sixty years. On his hundreth birthday in 1910, Dr. Jones received congratulations from King Edward. Total abstainers and non-smokers will doubtless note for future reference that he was of their company in both abstentions.

### TERRITORIAL DOCTORS RESIGN THEIR COMMISSIONS.

A new move in the opposition of the medical profession to the Insurance Act has now taken place at a meeting of the Cardiganshire Provisional Medical Committee which was held at Lampeter. Dr. Joshua Powell, of Newcastle Emlyn, presided, and a resolution was passed unanimously to support the movement that all medical practitioners acting as medical officers to the Territorial Force be called upon to resign their commissions. The main argument leading to this result was that as the State intended to cripple the medical profession through working the Insurance medical benefit, medical men could not be expected to serve the State by leaving their practice in order to go to camp on terms which meant a financial loss to them. It remains to be seen how far action on these lines will be taken, but the Government will hardly welcome any movement that tends to hamper the (rightly or wrongly) much maligned body which Lord Haldane has created so painfully.

### THE BACKSLIDING GUARDIANS OF LINCOLN.

The overcrowding of Lincoln Workhouse Infirmary, which, according to one speaker at a meeting last week, has been talked of for five years, has at last been authoritatively brought forward by an Inspector of the Local Government Board, the Hon. Gerald Walsh. "In winter," the Inspector remarked, "the infirmary is so much overcrowded that it is perfectly impossible to classify the cases or to provide for them in a proper manner." The question apparently has been postponed on the ground that only delay would prove whether it was an abnormal or a normal state of affairs. The Chairman of the Lincoln Guardians, Mr. J. Laverock, stated that they had been hoping to be relieved by the Legislature. Mr. Henry Smith remarked that they were quite conversant with the undesirable state of the infirmary, and had gone as far as to look out for a site for building a new institution. It was time they moved in the matter. Mr. W. G. Marshall and Mr. G. Harley urged that this was not the time for spending the ratepayers' money, whereupon Mr. W. Overton retorted that they would have to do so, whether they objected or not. A site had been selected, but had been "knocked out" by such objections as the above. Mr. Overton added that the Government architect had told him that they would not allow another brick to be laid so as to take away open space. There was, therefore, he argued, only one course left—namely, to build a new infirmary. We agree with him, and remind the Lincoln Guardians that the position is getting worse, and that the present deplorable condition which has made the Lincoln Workhouse Infirmary a symbol of inertia among Poor-Law institutions of the Birmingham type, whose advance (our Commissioner noted in an interview a week or two ago) is largely due to the backward policy of the last five years.

### THE LATE "GENERAL" BOOTH AND MEDICINE.

The death of the founder of the Salvation Army has drawn general attention to the diverse activities of the giant organisation he founded and controlled for so many years. One of the aspects of its work is much less in the public eye than some of the others. We refer to the useful work that the Army has done and is still doing in social hygiene. Its officers, male and female, are practical district nurses, and many doctors speak highly of their help, while everyone who has followed the personal career of the late General knows how much stress he himself has always laid on the importance of cleanliness and sobriety. Indeed, General Booth's "rule of life" is as well known as the tenets of his rival in old-age recipes, Professor Eli Metchnikoff. It is probable that in both cases the regimen so highly vaunted has had little to do with the length of years both renowned suggestors have achieved. Professor Metchnikoff's hypothesis is still on trial, and he is fortunately still alive. General Booth died a ripe octogenarian, but his longevity is not startling, for many men who lived far less carefully lived longer lives. As a matter of fact, longevity is probably dependent on factors altogether different from diet and regimen, the notable example of Cornaro notwithstanding. Perhaps Professor Pearson may subject the matter to critical statistical analysis; such an investigation should at least be interesting and may be highly instructive as well. General Booth, while not actively interested himself in the advance of modern medicine, was keen to take advantage of its progress. Salvation Army workers in foreign fields combined in many cases amateur medical with social and religious work, often most successfully. Nor must it be forgotten that the work which the Army has carried on in establishing homes for women and shelters for men is in a sense social hygienic work, which has tended to improve the physical fitness of the class among which it was carried on. As hospital visitors Army officials have also done good work. We do not for a moment suppose that there is likely to be any cessation in this social hygienic work now that General Booth is dead.

### A CURIOUS SUGGESTION FROM PONTYPOOL.

Mr. George Jenkins, vice-president of the Pontypool and District Hospital, presided at the recent half-yearly meeting of governors in place of Mr. A. A. Williams, the senior vice-president, whose great services in attending committee meetings and in maintaining interest in the institution from the beginning were enthusiastically referred to. Mr. Tom Langley, of Pontnewynydd, asked a question with reference to an item of expenditure debited to "surgery and dispensary." He thought that every doctor should provide himself with drugs at the hospital. Mr. Jenkins replied that drugs and dressings were provided at every hospital. Mr. Udell asked if the doctors gave their services gratis, and the Chairman agreed with another speaker who said that if private patients were treated at the hospital the fee which they paid went not to the doctors but to the hospital's funds. The auditors congratulated the Secretary, Mr. Tom Morgan, on the way in which the accounts were kept, and it would be interesting to know whether the highly successful "comic football match" was his idea also. Mr. Langley's argument for forcing doctors to provide their own drugs did not gain any support from the meeting.

### COMPULSORY HOSPITAL SUBSCRIPTIONS.

The North Lonsdale Council has received a deputation from the trade organisations of Barrow-in-Furness, headed by Councillor C. Ellison, asking that the representation of working men on the committee of the hospital should be in proportion to the amount subscribed by the workmen in the various works. Mr. J. J. Waddington, secretary of the hospital, has written in reply stating that the council of the hospital will submit the whole question of representation to the annual meeting of subscribers. The conference of delegates of the trade-union branches has demanded a special general meeting to consider its proposals. At the same conference a letter was also read from a trade union objecting to the Town Council's practice of making subscriptions to the hospital a condition of service for municipal employees. These facts are enough to show that the relation of the hospital to the working men is becoming a very strained one, and as such a strain is very harmful to the interests of either party we hope that every consideration will be given to the workmen's proposals, so that a more amicable feeling may speedily be brought about.

### THE LAW'S DELAY AND CORBETT HOSPITAL.

The medical staff of the Corbett Hospital, Stourbridge, pleaded at the recent annual meeting for increased accommodation. They were warned by Viscount Cobham, who presided, that "the present was hardly the time when you can appeal for widespread and large contributions." He added, however, that some day there would be a large sum of money to be distributed by the trustees of the Corbett Estate, of which he is one, and he hoped that some might go in this direction. But he quickly dashed this incipient hope to the ground by adding: "The trustees are not any nearer to the realisation of a divisible surplus than they have been for some years past." Such vague hopes are almost as bad as disappointments, and it is a pity if in his double capacity as president of the Corbett Hospital and as trustee of the Corbett Estate, Viscount Cobham cannot do something more than lament the law's delay in this important matter.

### SEA-WATER FOR INFANTILE DIARRHŒA.

It has been known for a long time, both by specialists and general practitioners, that the subcutaneous injection of sterile normal saline solution is a remedy of the utmost value in dealing with the collapsed condition of children suffering from acute epidemic diarrhœa. Although this fact was stated in several books, it had somehow not been appreciated by the mass of the profession in this country, although a few have practised the method for a long time. Consequently the egregious "Quinton theory," with its fantastic assumptions concerning the virtues of sea-water, impressed some clinicians because the treatment certainly did good in some cases. It was, however, not overlooked by the thoughtful that Quinton's diluted sea-water, retailed at an exorbitant price, was of much the same strength as ordinary normal saline, and this naturally led to increased attention being paid to the old-established virtues of the latter. Dr. J. R. Mackenzie has carried out clinical experiments with a view to ascertaining whether, in fact, normal saline solution is as effective as the much advertised sea-water in the treatment of acute enteritis in infancy. To this question, however, he does not provide any definite answer; but he does show the very rapid and striking benefit which normal saline, and even pure distilled water, will cause. Both these act speedily and effectively, but the duration of the improvement is longer in the case of saline solution than in that of distilled water. He says the latter should be injected subcutaneously at the earliest indications of collapse, and in the case of very young infants at the first visit, whether collapsed or not.

### A LIVERPOOL LADY AND THE COUNTRY HOSPITAL.

The Liverpool Country Hospital for Children has just received £2,000 from Miss Fowler for endowment purposes. This is interesting because it is not the first occasion on which Miss Fowler has generously endowed beds at the institution. Two cots have previously been set permanently free for patients by her generosity. On previous occasions we have drawn attention to the valuable work done by this hospital and the open-air wards in which the treatment is carried out. As a pioneer institution on its own lines it is gratifying to see its work recognised in this consistent manner by at least one subscriber.

### A PRACTITIONER OF THE OLD SCHOOL.

The death of Mr. William Clapton, F.R.C.S., at Canterbury, at the age of seventy-eight, removes a surgeon whose activities extended over many fields. He received his education as a boy at Christ's Hospital, and then became a student of St. Thomas's Hospital in its old quarters at London Bridge, where his brother, Dr. Edward Clapton, had been educated before him. After qualifying, he went into practice in the City, and represented for ten years the Vintry Ward on the Court of Common Council, a post from which he retired in 1882. On retiring from practice he settled at Canterbury, where his strong Churchmanship and social proclivities indulged themselves in many forms of personal service and voluntary charity.

### A NEW ACT IN OPERATION.

An inquiry which we believe to be the first of its kind under the Housing and Town Planning Act is now being held in North Wales at Abergele. There is an acute dispute in progress between a Mr. F. J. H. Bowdage and the local Urban District Council concerning some house property. It is remarkable that, though this property is described as of modern construction, the demands of the sanitary committee include a formidable number of requisitions, which point to the conclusion that the inhabitants of these houses cannot be enjoying any considerable comfort during the present meteorological conditions. Among these demands we find the owner called upon to provide separate water-taps for each of the houses, to cure external dampness, and to make good dry-rot in the floors. Great interest is displayed locally in the inquiry, and we understand the owner intends to fight the Council to the bitter end. The matter is one which will be followed closely by all local governing bodies, as it is the first inquiry held by the Local Government Board, who have sent down Mr. Edward Leonard to adjudicate upon it.

### THE CLERGY'S OBJECTION TO HOSPITAL CONCERTS.

The clergy and ministers of religion in the borough of Dewsbury have sent a letter to the authorities of the Dewsbury and District Infirmary protesting against a Sunday concert which was held at seven o'clock on July 14 in Crow Nest Park on behalf of the funds of the institution. The reasons of the protest were twofold—first, as to the nature of the music; and, secondly, that the time it was performed was "during the hours commonly set apart for religious worship." Mr. S. Fitton, the president, stated at the monthly meeting at which this letter was read that the Board had nothing to do with the arrangement, for which apparently the Sunday Concert Committee was responsible. The letter was then taken in committee, and a resolution was unanimously passed recommending the Concert Committee not to arrange any Sunday concerts on behalf of the infirmary during service time, and to put on the programme music "in keeping with the occasion." It is natural for the Dewsbury and District Infirmary board to wish not to offend the susceptibility of the local ministers, but movements to prevent any form of public recreation to take place at 7 p.m., the recognised time for all forms of assembly, have made the Churches look too like a vested interest to commend them to public regard. The surest way to prevent people from going to church is to attempt to compel them to go. Should any one body have a monopoly of 7 p.m.?

### DISPENSARY APPOINTMENTS IN SHEFFIELD.

The Sheffield City Council has completed arrangements with the Insurance Committee for the City for the treatment of insured persons. This will include hospital treatment at the Crimicar Lane, Common Side, and Winter Street institutions, tuberculin treatment at the dispensary, and home treatment. It must be remembered that Sheffield possesses already a tuberculosis dispensary, with a whole-time officer and a staff of men and women inspectors. This staff will be increased by the appointment of a second dispensary medical officer and an additional man inspector. Dispensary work, on its modern lines, as that not merely of a treatment centre but one for sifting the cases, is, like the hospital almoner's, full of interest and of great potentialities. It is, therefore, to be expected that numerous candidates will offer themselves for all such additional posts as the working of the sanatorium benefit may necessitate.

### A NEW BLOCK AND ITS PROBLEMS.

Since the new ward has been built at the Coventry and Warwickshire Hospital the need for more ground round the institution has become very apparent. At a meeting of the general committee last week, it was announced that the hospital's application some months ago to the Corporation for the purchase of a strip of land at the back of the mortuary will be favourably considered shortly. It has now been found, however, that still more land is really needed, and a further application to the Corporation has been made. In supporting this the Chairman, Mr. E. M. Iliffe, said that their first application ought really to have been for a larger site, but the building of the new ward had emphasised the congested area, and they now proposed to get as much land as the Corporation would give them at a reasonable price. In the meantime the fitting of the new block for sanitary and kitchen purposes has been taken in hand, and the Secretary, Mr. Rudd, together with the matron and the architects, has been inspecting various makes in London. They found, naturally, that the fittings of different firms varied very much in pattern, and this made an exact estimate difficult to secure. Still, all the chief contracts in connection with the extensions have now been placed. The new block is thus seen to have involved many matters not foreseen at the time it was decided on, and the land question is a good example of the care that should precede the erection of any hospital extension.

### PHARMACY, PHOTOMICROGRAPHY, AND FINANCE.

The many outside interests which pharmacists manage to combine with institutional work are well shown by such facts as the article we publish this week on photomicrography and the recent contribution of Mr. H. C. T. Gardner, M.P.S., to *The Financier and Bullionist* on "The Trade in Drugs and Chemicals." Mr. Gardner, who is well known amongst institution dispensers as pharmacist at the Miller Hospital, Greenwich, has achieved many successes, evidently taking his occupation very seriously. Besides writing financial and expert articles for the scientific and lay press—being especially interested in rubber and its chemistry—he has read papers, both of his own compilation and in collaboration, before various scientific societies. One of the latest was entitled "The Therapeutic Uses of Radium," which he and Mr. O. A. Elias, F.C.S., prepared for a meeting of the Pharmaceutical Society. He was chairman of the Public Pharmacists' Association for three successive years, 1906, 1907, and 1908. Mr. Gardner is a Fellow of the Chemical Society.

### "SYNDICALISM" IN MEDICINE.

In refusing to resign his seat on the Chester Insurance Committee at the request of the British Medical Association, Dr. T. S. Parry, M.B., says: "I believe in both parties, Government and medical profession, making the best bargain they can by reasonable and fair negotiation . . . . By declining any further negotiation . . . the medical profession might succeed in wrecking the Act. By the same tactics they might proceed to dispose of the Government as well; the machinery is the same, it is only a variation in the direction of its energy. This method of employing the powers of a monopoly, I believe, is called 'Syndicalism,' and is the method promulgated by Ben Tillett and Tom Mann. I believe heart and soul in the Insurance Act." Dr. Parry is mistaken: Syndicalism has nothing to do with employing a monopoly as a method of attack, it is a political theory meaning the vesting of every industry in the workers of every industry. Apart from this, however, we have nothing to add to our comments on the non-compliant medical men that we published last week. Dr. Parry's post under the Insurance Act is the double one of member of the Chester Insurance Committee and chairman of the Sanatorium Sub-Committee. He and Mr. Griffith, another Chester medical man, who has refused to resign, are both well-known local Liberals.

### PORTSMOUTH HOSPITAL HONOURS AN OLD HOSPITAL WORKER.

The late Dr. Claude Claremont was associated with the Royal Portsmouth Hospital for more than thirty years, and his death last year deprived the institution of a good friend and ally. Beginning his association as house surgeon shortly after his qualification, Dr. Claremont continued it in the capacity of out-patient physician, assistant physician, and physician, and as such did good work, both for the patients and for the hospital. He served on important committees, and his experience, skill, and tact did much to ensure cordial relations between the various branches of the hospital. The Royal decided, shortly after his death, to commemorate his services by some simple monument. Last month this was unveiled in the main corridor of the institution. It consists of a fine memorial tablet of Sicilian marble in a gilt mosaic frame. The tablet is suitably inscribed, and is the gift of Dr. Claremont's colleagues and personal friends.

### HOSPITAL LABORATORY BOYS.

The death of Dr. John Wade, Professor of Chemistry at Guy's Hospital Medical School, removes one of the best known and most popular of the members of Guy's staff. Dr. Wade's career should prove an inspiring history to all laboratory assistants, for he started from the bottom of the ladder, and by sheer pluck and self-help became chief of the laboratory in which he had worked as an ordinary assistant. He was never ashamed of his early days, and gave sympathy and assistance to everyone who worked with him, though he had the reputation of being an exacting teacher and chief. It is remarkable that Guy's has seen the rise of several laboratory boys who have attained to eminence through similar effort and persistent hard work. The name of Sir William Gull, the poor boy who was befriended by a hospital treasurer, and from bottle-washer rose to become one of the foremost specialists in England, stands easily first on the list. But it is a sad fact, notwithstanding these brilliant exceptions, that ward or laboratory work for boys is almost as much a blind-alley occupation as is van work. The "lab. boy" learns little that is useful in a practical way when he is dismissed for what in the laboratory amounts to old age. And he finds it difficult to obtain employment afterwards at a rate equal to that which he received in the school. This is a matter which might well engage the attention of hospital authorities.

### LADY RESIDENT DOCTORS AT POOR-LAW INFIRMARIES.

The Dewsbury Board of Guardians, at a recent meeting of which Mr. George Blacker presided, proposed to appoint a lady resident doctor at the workhouse infirmary. The Local Government Board, however, regards the proposal as inexpedient, and states that it is not aware of any case in which an institution of the size of that at Dewsbury has been placed in charge of a woman as principal medical officer. The Board also said that, if the experiment of appointing a woman were to be made, satisfactory arrangements were necessary for the examination and treatment of cases with which a woman could not properly deal. The Deputy Clerk, Mr. E. T. Tunnicliffe, said that it was proposed also to appoint a consulting surgeon. Three Guardians, Mr. J. H. Dyson, Mr. Appleyard, and Mr. Newsome, were in favour of appointing a woman resident and a visiting surgeon. The matter was eventually adjourned pending further inquiry.

### HOSPITALS AND THE VINE.

The famous vine at Hampton Court is not the only phenomenal vine near London, nor the only one many of whose grapes eventually find their way into the London hospitals. Near Cumberland Lodge, Windsor Great Park, is another great vine, the grapes of which are said to be finer than those at Hampton Court. This year it is bearing some 500 bunches, some of which follow the King and Queen wherever they happen to be staying, while those bunches which are not required are sent to the hospitals. This year many of the bunches are over 4 lb. in weight, and the vine itself is 120 feet long, 20 feet wide, and almost 150 years old. Its care demands the whole time of one gardener. We hope that the patients who are lucky enough to receive the fruit this year will enjoy it all the better for being reminded of these facts.

### THIS WEEK'S DRUG MARKET.

Business in the Drug market is still without improvement, and few price-changes of importance have taken place. Following on a reduction in the value of quicksilver makers of mercurials have slightly reduced their quotations for calomel, corrosive sublimate, and other salts of mercury. Opium is rather dearer, and in consequence the price of morphine tends upwards. Rather high prices were quoted for English oils of peppermint and lavender. At the recent public sale of drugs in Mincing Lane there was a better demand, a good proportion of the offerings being sold.

